# Correlates of current cigarette smoking among in-school adolescents in the Kurdistan region of Iraq

**DOI:** 10.1186/1752-1505-1-13

**Published:** 2007-12-04

**Authors:** Seter Siziya, Adamson S Muula, Emmanuel Rudatsikira

**Affiliations:** 1 University of Zambia, School of Medicine, Lusaka, Zambia; 2 Department of Community Health, University of Malawi, Blantyre, Malawi; 3 Departments of Global Health, Biostatistics and Epidemiology, School of Public Health, Loma Linda University, California, USA

## Abstract

**Background:**

Many adult cigarette smokers initiated the habit as adolescents. Adolescent tobacco use may be a marker of other unhealthy behaviours. There are limited data on the prevalence and correlates of cigarette smoking among in-school adolescents in Iraq. We aimed to estimate the prevalence of, and assess the socio-demographic correlates of current cigarette smoking among in-school adolescents in Kurdistan region of Iraq.

**Methods:**

Secondary data analysis of the Global Youth Tobacco Survey, conducted in the region of Kurdistan, Iraq in 2006. Logistic regression analysis was conducted to assess the association between current cigarette smoking and explanatory variables.

**Results:**

One thousand nine hundred eighty-nine adolescents participated in the Kurdistan-Iraq Global Youth Tobacco Survey. Of these, 58.1% and 41.9% were boys and girls respectively. The overall prevalence of current cigarette smoking was 15.3%; 25.1% and 2.7% in boys and girls respectively. The factors associated with adolescent smoking were: parents' smoking, smoking in closest friends, male gender, having pocket money and perceptions that boys or girls who smoked were attractive.

**Conclusion:**

We suggest that public health interventions aimed to curb adolescent cigarette smoking should be designed, implemented and evaluated with due recognition to the factors that are associated with the habit.

## Background

Tobacco use is a leading cause of morbidity and mortality from non-communicable diseases globally [1,2]. Barzani has reported that the top five causes of mortality and morbidity in Kurdistan-Iraq are cardiovascular disease, cancer, respiratory disease, and hypertension, many of these associated with smoking [3].

Over the past 10 years, there has been growing research interest to estimate the prevalence of tobacco use among adolescents. This effort has largely been driven by the Global Youth Tobacco Survey Collaborating Group as part of the Global Tobacco Surveillance System initiated by the World Health Organization (WHO), CDC, and the Canadian Public Health Association.

Adolescent cigarette smoking is of public health significance. Many adult smokers had initiated the habit as adolescents. Also smoking among adolescents has short to medium term health effects in the smokers as well as peers who may be exposed to environmental smoke. Smoking in adolescents may also be a marker of other harmful lifestyles such as engagement in illicit drug use, alcohol use, psychiatric illnesses and sexual intercourse [4-6].

The Centers for Disease Control and Prevention (CDC) has reported on the prevalence of tobacco use including cigarette smoking among in-school adolescents in Kurdistan, Iraq [7]. This study reported on the analysis of the Kurdistan-Iraq Global Youth Tobacco Survey. The study reported that 11.9%) were current cigarette smokers. Boys were significantly more likely than girls (21.0% versus 2.1%) to smoke cigarettes currently. Current smoking was defined as having reported smoking cigarettes, even a single puff, within the last 30 days of the survey.

The CDC's paper reported on prevalence of tobacco use and other social characteristics among in-school. This paper restricted data analysis to 13 to 15 year olds and did not assess the socio-demographic correlates of being a current cigarette smoker. While estimates of prevalence of tobacco use will certainly quantify the burden of the problem, it is not possible to identify factors that are associated with cigarette smoking without a deliberate assessment of the potential correlated. We therefore carried out a secondary analysis of the Kurdistan-Iraq Global Youth Tobacco Survey to identify the factors that are associated with current cigarette smoking among in-school adolescents in this region of Iraq.

## Methods

### Study setting

The Kurdistan-Iraq Global Youth Tobacco Survey was conducted in the region which covers the governorates of Irbil, as-Sulaymaniyah, and Dahuk. The Kurdistan region borders Iran to the east, Turkey to the north and Syria to the west. Its capital is the city of Erbil.

### Study participants recruitment

The GYTS is a cross-sectional survey which aims to collect data from 13 to 15 year old in-school adolescents. Although the GYTS aims to collect data from 13 to 15 year olds, all students in the selected classes are invited to participate. In the Kurdistan region of Iraq, this age range is covered by the first through fourth years of secondary education. The GYTS uses a cross sectional two-stage sampling design. In the first stage of sampling, the sampling frame included all schools containing secondary school grades 1 to 4. The probability of a school being selected was proportional to the enrolment size of the school in the selected grades. In the second stage of sampling, classes within the selected schools were selected randomly. All students present in the classes that had been selected were eligible to participate in the survey. There was no age restriction once a class had been selected. A total of 1,989 students completed the GYTS (58.1% male and 41.9% female). The school response rate was 100% (25 schools), the student and overall response rates was 95.6%.

### Data collection

The data from the Kurdistan-Iraq GYTS (2006) were collected anonymously from the eligible students who were present on the day of the survey at their school. A self-completed, computer scannable questionnaire was used. Students were asked questions about their smoking practices, socio-demographic information and exposure to tobacco-related media.

### Data analysis

For the purpose of the current analysis, we were interested in the prevalence and factors associated with current cigarette smoking. Current cigarette smoking (the main outcome) was defined as self-report of having smoked a cigarette, even a single puff, within the last 30 days. No biomarkers were used to verify the self reports. We also assessed the distribution of social characteristics among the study population. These characteristics included: gender, age, amount of pocket money usually received each month, parental smoking, smoking among closest friends, exposure to tobacco-related media and perceptions towards smoking.

A weighting factor was used in the analysis to obtain prevalence estimates to reflect the likelihood of sampling each student and to reduce bias by compensating for differing patterns of non response.

We also conducted weighted backward logistic regression analysis using SPSS software version 14.0 (Chicago, Illinois, United States of America) to assess what relationship existed between a selected list of explanatory variables and the main outcome. The explanatory variables were identified from the literature as having been associated with adolescent smoking in other settings. These variables included: gender, age, parental smoking, smoking in friends, exposure to tobacco-related media, perceptions towards tobacco and the amount of pocket money at the disposal of the adolescent [8-12].

## Results

### Characteristics and prevalence of smoking among study participants

One thousand nine hundred eighty-nine adolescents participated in the Kurdistan-Iraq Global Youth Tobacco Survey. Of these, 58.1% and 41.9% were boys and girls respectively. The overall prevalence of current cigarette smoking was 15.3%; 25.1% and 2.7% in boys and girls respectively. Further description of the study participants is shown in Table 1.

**Table 1:** Characteristics of study participants in the Iraqi Kurdistan Global Youth Tobacco Survey 2006

Demographic factor	Total	Male	Females
Age (years)			
17+	342 (19.2)	251 (26.6)	89 (9.2)
16	332 (16.7)	159 (16.5)	169 (17.1)
15	372 (18.8)	187 (18.6)	183 (19.4)
14	296 (15.3)	128 (13.0)	164 (18.6)
13	289 (14.7)	125 (13.0)	160 (17.0)
12	211 (10.3)	65 (7.1)	139 (14.4)
<12	98 (5.1)	51 (5.3)	41 (4.3)

Sex			
Male	982 (58.1)		
Females	969 (41.9)		

Schooling grades			
Fourth	278 (13.2)	80 (12.3)	192 (14.2)
Third	442 (22.9)	244 (22.6)	171 (23.4)
Second	555 (28.6)	304 (28.8)	243 (28.6)
First	709 (35.3)	352 (36.4)	344 (33.7)

Pocket money (Iraqi Dinars)			
30000+	142 (7.7)	93 (9.8)	47 (5.0)
21000–29999	107 (5.6)	65 (6.7)	42 (4.4)
11000–20999	114 (6.1)	73 (7.4)	41 (4.4)
6000–10999	191 (9.7)	110 (11.2)	80 (8.0)
1000–5999	275 (14.5)	155 (16.2)	116 (12.4)
<1000	262 (13.4)	118 (12.2)	137 (14.6)
None	858 (43.0)	352 (36.6)	485 (51.2)

Current smoker			
Yes	255 (15.3)	225	27 (2.7)
No	1615 (84.7)	675 (74.9)	914 (97.3)

### Socio-demographic factors associated with current cigarette smoking

Table 2 shows multivariate analysis to identify the association between current cigarette smoking and the selected variables. We found that adolescents >15 years were more likely to be smokers compared to younger study participants. Overall, some pocket money, male gender and lower school grades were associated with smoking.

**Table 2:** Demographic and economic factors associated with current smoking among adolescents in Kurdistan, Iraq, 2006

Demographic factor	OR (95% CI)
Age (years)	
17+	1.93 (1.86, 2.00)
16	1.30 (1.25, 1.35)
15	1.07 (1.03, 1.11)
14	0.62 (0.59, 0.65)
13	0.49 (0.46, 0.51)
12	0.43 (0.41, 0.46)
<12	1

Sex	
Male	2.21 (2.15, 2.27)
Females	1

Schooling grades	
Fourth	0.84 (0.81, 0.87)
Third	0.94 (0.92, 0.97)
Second	0.70 (0.67, 0.72)
First	1

Pocket money (Iraqi Dinars)	
30000+	0.69 (0.66, 0.73)
21000–29999	1.04 (0.99, 1.09)
11000–20999	2.45 (2.35, 2.56)
6000–10999	1.44 (1.38, 1.49)
1000–5999	1.21 (1.17, 1.26
<1000	1.03 (0.99, 1.07)
None	1

Also as shown in Table 3, we found that having a parent who was smoker (father or mother), having most or all of one's closest friends as smokers, perception that 1) boys who smoke have more friends, or 2) that a boy or girl who smoke was more attractive, were associated with being a current smoker.

**Table 3:** Social factors associated with current smoking among adolescents in Kurdistan, Iraq, 2006

Demographic factor	OR (95% CI)
Parents smoke	
Father only	1.14 (1.10,1.18)
Mother only	1.65 (1.55,1.77)
None of them	1

Closest friends smoke	
All of them	3.83 (3.66,4.00)
Most of them	2.37 (2.29,2.45)
Some of them	0.64 (0.63,0.66)
None of them	1

Boys who smoke have more or less friends	
More friends	1.32 (1.28,1.35)
Less friends	0.88 (0.86,0.90)
No difference from smokers	1

Girls who smoke have more or less friends	
More friends	0.60 (0.58,0.62)
Less friends	0.82 (0.80, 0.85)
No difference from smokers	1

Smoking cigarettes makes boys look more or less attractive	
More attractive	1.15 (1.12, 1.18)
Less attractive	0.69 (0.67,0.71)
No difference from non-smokers	1

smoking cigarettes makes girls look more or less attractive	
More attractive	1.26 (1.22,1.30)
Less attractive	1.09 (1.06, 1.12)
No difference	1

We also assessed whether exposure to various sorts of media was associated with being a current smoker. As shown in Table 4, the analysis produced mixed results. We found that being exposed to a lot of tobacco advertisements at sports events, concerts and fairs and having seen a lot of movie actors smoking in movies or videos were associated with being a cigarette smoker. However, exposure to tobacco advertisement from billboards and magazines were not associated with being a smoker. In fact, being exposed to a lot of billboard and magazine tobacco advertisements was found to be protective from being a smoker.

**Table 4:** Associations of factors related to advertisements for smoking with current smoking among adolescents in Kurdistan, Iraq, 2006

Demographic factor	OR (95% CI)
Frequency of seeing actors smoke when watching television, videos or movies	
A lot	1.14 (1.11,1.17)
Sometimes	1.01 (0.98,1.04)
Never watched	1

Frequency of tobacco advertisements seen on billboards	
A lot	0.80 (0.78,0.83)
A few	1.30 (1.27,1.34)
None of them	1

Frequency of advertisements or promotions for cigarettes seen in newspapers or magazines	
A lot	0.90 (0.87, 0.93)
A few	1.01 (0.99,1.04)
None of them	1

Frequency of advertisements or promotions for cigarettes seen at recreational events	
A lot	1.61 (1.57,1.66)
Sometimes	0.94 (0.91, 0.96)
Never watched	0.75 (0.73,0.78)
Never	1

## Discussion

The prevalence of current cigarette smoking among the study participants in the Kurdistan-Iraq Global Youth Tobacco Survey was 15.3%, higher among boys compared to girls (25.1% versus 2.1%). This prevalence is much higher than the 10.4% reported by Kyrlesi among Greek adolescents 13 to 15 years [13]. Our estimates are also slightly different from those reported by the Centers for Disease Control [7] because in this previous analysis, analysis was only limited to 13 to 15 year olds. This previous study also did not assess the association between any explanatory variables and self-reported history of smoking.

Current cigarette smoking among Ethiopian in-school adolescents was 4.5% among boys and 1.0% among females in 2003 [10]. Our estimate of Kurdistan-Iraq adolescents is however much lower than has been reported for 16 to 18 year old Israeli adolescents (overall prevalence 25.7%, 26.2% in males and 21.1% among females respectively) [14].

Maziak et al reported on prevalence of cigarette smoking among university students in Syria [15]. In this study, where study participants were much older (mean age 21.8 years) that in the Iraq GYTS, 30.9% males and 7.4% females were smokers. In a sample of Turkish youths (mean age 17.6%), the percentage of current smokers reported by Aras et al (2007) in males was 30.4% compared to 17.4% among females [16]. The estimates reported by Aras et al are much higher than what we have reported in this study.

Our study found that current smoking prevalence was higher among boys compared to girls (25.1% versus 2.1%). The male predominance in cigarette smoking has been reported elsewhere [10,13-17]. However fewer studies have reported why the gender disparity occurs. Maziak et al [18,19] have explored the reasons why most women in Syria were non-smokers. The reasons included religious convictions, perceptions about smoking among husbands, family values and traditions and limited economic resources. Family and societal values and traditions in Iraqi Kurdistan are more tolerant of smoking among boys that they are for smoking among girls. In 2002, Maziak [20] estimated that prevalence of current tobacco smoking was 48% among adult males versus 9% among women. Barzani has also reported that cultural and religious taboos towards smoking among women could be an important factor in Kurdistan-Iraq [3].

While male predominance in smoking has been reported in many settings, this finding is not universal. Steele et al had reported that girls were more likely to be current and former smokers in Minnesota, United States [21].

We also found that adolescents who had friends or parents who were smokers were also more likely to be smokers themselves. Similar findings have been reported elsewhere [9,11,12,22]. Parental smoking could influence the availability and access to cigarette by the adolescent as well as affect tolerance to smoking by the parent. Adolescents who have parents who are less tolerant to smoking are less likely to smoke themselves.

In the case of having friends who smoke as a factor associated with smoking, Simmons-Morton [23] and Hoffman et al [24] have reported that both selection of friends and socialization effects may play a role. In terms of selection and socialization of friends, adolescents who are already smokers may be more likely to choose other smokers. Peer influence may also affect initiation of smoking in adolescents who start off as non-smokers but have selected smoker as friends. Choosing a friend who is a smoker may also be a predictor of the adolescent's tolerance to smoking. Kim and Clark [25] have reported that adolescents who attended schools with higher student smoking rates; adolescents who had easier access to cigarettes at home; and adolescents who had more friends smoking were all more likely to be adverse transitioners from low and mild smoking to heavy smoking by young adulthood.

When we assessed whether exposure to tobacco related advertisements was associated with being a current smoker, we found inconsistent results; some analysis showed exposure positively associated while others negatively associated with smoking (See Table 4) The mixed findings in terms of exposure to pro-tobacco advertisements and adolescent smoking may have arisen due to unmeasured confounders. Due to the secondary data analysis nature of study, we were only able to work with the available variables. It is possible that other factors in the socio-cultural milieu of the study area may have resulted in pro-tobacco advertisements having the unintended effects. For example, if the wide availability of tobacco advertisements was influencing the provision of aggressive anti-tobacco interventions, even with adolescents having been exposed to these adverts, if the interventions were successful, more advertising may not reap the intended effect.

Although we found that being exposed to a lot of magazine and billboard tobacco advertisements was negatively associated with being a smoker, we would hesitate in recommending exposure to such media as a reasonable measure to curb adolescents. Our decision is based on the following. Firstly, there is ample previous evidence that positively link pro-tobacco advertisements and adolescent smoking. Secondly, our results may have come about due to failure to control for unmeasured confounders. It is possible that having access to magazines may be a marker of some other variable. Exposure to billboards may also be a marker of residence status and possibly other socio-economic status. We would however suggest that this issue about the relationship between exposure to tobacco related media and current cigarette smoking status be examined further in this setting. It is not adequate to always conclude that since pro-tobacco media advertisements had been associated with adolescent smoking, then similar findings may also be obtained in all settings.

We found that in general, the higher the amount of pocket money reported by the study participant, the more likely he or she was a smoker. Having access to pocket money may facilitate how an adolescent may access cigarettes. Unger et al have reported that having access to pocket money was associated with being a smoker among adolescents in the United States [26]. These authors suggested that limiting the amount of pocket money could be an effective strategy aimed to prevent adolescents smoking. Scragg et al [27] has also reported similar findings in New Zealand. We believe that having some disposable income was a factor in influencing adolescents' access cigarettes. However, our results did not show a strictly linear pattern.

Various initiatives aimed to prevent tobacco use among adolescents in the Kurdistan region of Iraq have been planned and established. Barzani [3] has reported that a tobacco-control has been recognized as a priority within the Ministry of Health. A unit on tobacco control has been established. Barzani also reports that Kurdistan the law bans smoking in all government buildings, although strict implementation of the law has not been possible.

As reported above, the Kurdistan Region has been an autonomous region of Iraq for over a decade. Although the region has, to a large extent been spared from the conflict in the rest of the country, there may still be factors that have affected the region stemming from the conflict in the rest of the country. These factors may or may not affect the prevalence of smoking in the region.

The government of the Regional Government of Kurdistan-Iraq has planned for anti-tobacco education campaigns to be conducted in schools throughout the Kurdistan region during the 2006–07 academic year. These will include displayed of antismoking posters and distribution of pamphlets. While Kurdistan law bans smoking in all government buildings, including schools and administrative office buildings of MOH and the Ministry of Education (MOE), strict adherence to these regulations has not been possible.

### Limitations of the study

The findings in this report are subject to some limitations. Firstly, the sample that we participated in the survey was limited to adolescents enrolled in school and present on the day of the survey. As such our findings may not be representative to all adolescents in the Kurdistan region of Iraq. We however believe that the findings are likely to be representative of the school-going adolescents as overall response rate was high i.e. 95.6%.

Secondly, the data were based on self-reports of students, who might have underreported or over-reported their behavior or attitudes. Brener et al [28] has reported high reliability of adolescent reports on their behaviours for responses to tobacco-related questions on United States surveys similar to the GYTS. It however remains to be determined whether the high reliability of findings from the United States is comparable to experiences in Iraq. The self reports on history of current smoking from study participants were not confirmed with relevant biomarkers.

We also examined the variables as operating at the individual level. However authors like Nichter [29] and Wilcox [30] have called for the examination of the interaction between individual-level variable and societal level variables' interactions.

Furthermore, our study was based on a secondary analysis of already existing data. We therefore did not have control of which variables to collect from the study participants. The literature on adolescent smoking has reported that other variables such sibling smoking status [31,32], alcohol use [33,34], and religiosity [35-37] may be important factors associated with adolescents. We suggest that future survey's collect such data.

While we have assessed the relationship between the amount of pocket money and being a smoker, we also realize that like in many developing and emerging markets countries, smuggling of tobacco products, including cigarettes, may be an important source of tobacco among adolescents [38-41]. There may therefore be different prices that an adolescent smoker may pay to access cigarettes dependent on the availability of smuggled, likely cheaper sources.

We are also unaware how the overall current conflict situation in Iraq may or may not have influence on the prevalence and associated factors of smoking in Kurdistan region of Iraq. We do recognize though that unlike the other areas of the country, this region has to a large extent been administered as an autonomous region of Iraq and has not been active geographic area of the conflict.

## Conclusion

We have estimated adolescent smoking prevalence as 15.3% with males having higher prevalence than females. Evidence-based public health intervention should make use of our knowledge of the factors that are associated with smoking in Iraqi-Kurdistan. We suggest that research be conducted to assess the effectiveness of the interventions that are being provided in the region to prevent adolescent smoking. Also future studies should consider measuring other variables such as alcohol use, sibling smoking status and religiosity to enable assessment of how these factors may be associated with smoking in Iraq.

## Abbreviations

CDC: Centers for Disease Control and Prevention

GYTS: Global Youth Tobacco Survey

WHO: World Health Organization

## Competing interests

The author(s) declare that they have no competing interests.

## Authors' contributions

SS: conducted the data analysis, participated in the drafting of manuscript.

ASM: participated in the interpretation of the results and drafting of the manuscript.

ER: participated in the interpretation of results and drafting of manuscript.
